# T_H_9 cells in anti-tumor immunity

**DOI:** 10.1007/s00281-016-0599-4

**Published:** 2016-11-10

**Authors:** Thaiz Rivera Vargas, Etienne Humblin, Frédérique Végran, François Ghiringhelli, Lionel Apetoh

**Affiliations:** 1 INSERM, U866, Dijon, France; 2 Faculté de Médecine, Université de Bourgogne Franche-Comté, Dijon, France; 3 Centre Georges François Leclerc, Dijon, France

**Keywords:** Adaptive immunity, CD4 T cells, Gene transcription, Cancer, Innate immunity, Immunomodulation

## Abstract

IL-9 was initially identified as a T cell growth factor with a potential oncogenic activity. Accordingly, IL-9 drives tumor growth in most hematological cancers. However, the links between IL-9 and cancer progression have been recently revisited following the discovery of T_H_9 cells. T_H_9 cells, which have been characterized in 2008 as a proinflammatory CD4 T cell subset that promotes protection against parasites and drives tissue inflammation in colitis, actually harbor potent IL-9-dependent anti-cancer properties in solid tumors and especially melanoma. While the molecular mechanisms underlying these observations are still being investigated, T_H_9 cells were demonstrated to activate both innate and adaptive immune responses, thereby favoring anti-cancer immunity and tumor elimination. Human T_H_9 cells have also been identified in cancer tissues, but their functions remain elusive. The present review aims to discuss the anti-cancer potential of T_H_9 cells and their possible clinical relevance for cancer immunotherapy.

## Introduction

In 1893, William Coley and Robert Koch reported that the injection of streptococcal cultures into cancer patients drove tumor regression [1]. This was actually the first indication that the activation of the immune system was able to lead to tumor elimination. The development of cellular immunology then led Burnet to propose in 1957 the cancer immunosurveillance hypothesis, which postulates that developing tumor cells trigger an immune reaction that drives their elimination by immune cells before forming a clinically detectable tumor. It remained however difficult to understand why despite the existence of spontaneous immune responses to tumor antigens, this reaction failed to control tumor growth. Subsequent studies unraveled that the immune cells could play a dual role in cancer. They can either prevent tumor growth by eliminating tumor cells or drive tumor progression by favoring the emergence of tumor clones resistant to anti-tumor immunity and creating tumor promoting conditions in the tumor microenvironment. The latter concept named cancer immunoediting illustrates the ability of immune cells to shape tumor progression. The relevance of cancer immunoediting was confirmed not only in preclinical studies but also in humans [2]. Indeed, most human tumors infiltrated with either regulatory or effector immune cells are, respectively, associated with a worse and more favorable prognosis as reviewed elsewhere [3].

CD4 T cells display a high degree of plasticity and the ability to differentiate into various effector and regulatory T cell subsets that express distinct transcription factors and secrete different cytokine panels. CD4 cells actively participate in shaping anti-tumor immunity. T_H_1 and T_H_2 cells were the first-defined T_H_ lineages by Mosmann and Coffman in 1986. T_H_1 cells are ascribed with anti-cancer functions, notably because these cells secrete high levels of IFN-γ, which prevents tumor angiogenesis, enhances tumor cell immunogenicity by upregulating MHC class I and II expression, promotes recruitment of immune cells including CD8 T and NK cells that mediate tumor-killing activity, and increases the anti-tumor activity of macrophages [4–6]. Conversely, T_H_2 cells are associated with the promotion of tumor growth. This activity is primarily due to their secretion of IL-4 and IL-13 cytokines, which enhance tumor cell survival [7, 8]. Nevertheless, this initial dichotomy in T_H_ subsets failed to explain the complexity of CD4 T cell responses and the existence of additional CD4 T helper subsets was investigated, ultimately leading to the identification of other T_H_ subsets, including regulatory T cells (Tregs), T_H_17 cells, and Tr1 cells. Tregs and Tr1 cells suppress immune responses, favor the maintenance of immune tolerance, and contribute to tumor progression in the vast majority of cancers [9, 10]. The role of T_H_17 cells in cancer progression is controversial [6, 11]. T_H_17 cell-derived IL-17A favors tumor progression and neoangiogenesis. In addition, IL-17A triggers STAT3 activation in tumor cells, thereby favoring tumor growth [12]. However, in mouse models of melanoma, it was conversely shown that the adoptive transfer of T_H_17 cells could restrain tumor progression [13, 14]. The complex relationships between T_H_17 cells and cancer progression are further illustrated by observations in humans showing that T_H_17 cell infiltration is detrimental in colon cancer but beneficial in ovarian cancer [11]. Thus, CD4 T cell subsets and their associated cytokines can have profound effects on tumor progression.

In 2008, one novel CD4^+^ T cell subset secreting high levels of IL-9 was characterized and named T_H_9 cells. This CD4^+^ T cell subset was simultaneously discovered by two different laboratories [15, 16]. Veldohen et al. showed that TGFβ was able to reprogram the differentiation of T_H_2 cells into IL-9 producing T cells, while Dardalhon et al. demonstrated that IL-4 is able to block Foxp3 induction in Treg cells, thereby inducing a population of T helper cells that predominantly produce IL-9 [15, 16]. The discovery of T_H_9 cells thus further illustrates the plasticity among CD4^+^ T cells. The role of IL-9 in cancer has been previously explored. By generating transgenic mice overexpressing the *Il9* gene, Renauld and colleagues found that a small proportion of IL-9 overexpressing mice developed thymic lymphomas, suggesting that IL-9 supports the development of T cell tumors [17]. This observation was actually in line with the previously ascribed activity of IL-9 as a T cell growth factor [18]. IL-9 was then shown to promote the development of many hematological human tumors, including Hodgkin’s lymphoma and B cell lymphoma [19]. In addition, IL-9 was proposed to enhance the immunosuppressive functions of Tregs and to block the establishment of adaptive anti-tumor immunity by preventing the development of immunologic memory [20, 21].

While the aforementioned findings suggest that IL-9 can drive tumor progression, several investigators found that T_H_9 cells harbored anti-cancer properties in solid tumors, including lung adenocarcinoma and melanoma. Importantly, these anti-cancer properties were found to depend, at least in part, on T_H_9 cell-derived IL-9. In addition, T_H_9 cells were identified in human melanoma skin lesions, suggesting that they could possibly contribute to cancer immunosurveillance in this disease. In this review, we discuss recent findings that provide strong impetus to revisit the links between IL-9 and cancer progression and highlight the relevance of modulating T_H_9 cell functions for cancer immunotherapy.

## T_H_9 cell-driven activation of innate anti-cancer immunity

The seminal investigation on the role of T_H_9 cells in cancer was carried out by Purwar and colleagues who investigated the anti-tumor properties of T_H_9 cells in a mouse model of melanoma. Specifically, they tested the ability of tumor-specific CD4 T cells polarized into T_H_9 cells or other effector CD4 T cell subsets to prevent tumor outgrowth in B16 tumor-bearing mice upon adoptive transfer. They found that T_H_9 cells were highly efficient in preventing tumor progression in this setting. Importantly, the anti-cancer efficacy of T_H_9 cells was superior to all other CD4 T cell subsets tested, including T_H_1 and T_H_17 cells [22]. Upon studying the mechanism responsible for the anti-tumor activity of T_H_9 cells in melanoma, the authors found, in contrast to published studies in hematological cancers, that IL-9 blockade using neutralizing antibodies prevented the beneficial effect of adoptive T_H_9 cell transfer, underscoring the anti-tumor role for IL-9 in this setting.

The role of IL-9 in preventing melanoma cell growth was further explored in IL-9 receptor-deficient mice, and it was found that B16 tumor cells featured faster growth in vivo in the absence of IL-9 receptor signaling. Conversely, injection of recombinant IL-9 protein into wild-type mice impaired B16 tumor cell growth in vivo [22]. Interestingly, the anti-cancer effect of IL-9 was not restricted to melanoma as injection of recombinant IL-9 protein into Lewis lung carcinoma tumors also limited cancer growth [22]. Because IL-9 was not affecting melanoma or lung carcinoma cell proliferation in vitro, Purwar and colleagues have investigated whether host immune cells were responsible for the anti-cancer effect of IL-9 in vivo*.* Authors first tested whether the anti-tumor efficacy of T_H_9 cells was dependent on T cell immune responses from the host upon adoptive transfer. For this, they injected T_H_9 cells into tumor-bearing Rag1-deficient mice, which lack T and B cells, and found that the anti-tumor potential of T_H_9 cells was conserved in the absence of adaptive immunity. It is noteworthy that these results are supported by another study showing that the regulation of T_H_9 cell differentiation by the transcription factor Id3 regulated anti-melanoma immunity in an IL-9-dependent manner but without affecting T_H_1 cell responses [23]. In line with this, the anti-tumor effects of recombinant IL-9 administration were conserved in tumor-bearing Rag1-deficient mice, suggesting that other immune effectors are involved in the anti-cancer effects observed.

**Fig. 1:**
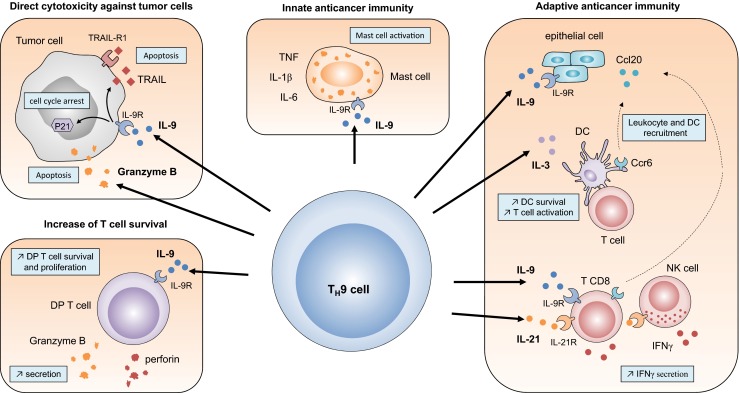
Pleiotropic effects of T_H_9 cells in anti-tumor immunity

IL-9 has been previously shown to trigger mast cell activation [24]. To study the contribution of mast cells to the anti-cancer effects triggered by IL-9 administration in vivo, the authors treated LLC1 and B16 tumor-bearing kit W-sh mice with IL-9 and found that the anti-tumor effects of IL-9 relied on mast cells in both tumor models [22, 25]. The role of mast cells in mediating T_H_9 cell-dependent anti-tumor immune responses was further investigated in an elegant study from Abdul-Wahid et al., who interrogated the cellular bases accounting for the anti-tumor efficacy of a vaccine containing the carcinoembryonic antigen (CEA) IgV-like N domain and the toll-like receptor 3 ligand poly I:C that elicited T_H_9 cell responses in vivo. In this setting, the importance of T_H_9 cell responses in mediating anti-tumor immunity following vaccination of CEA transgenic mice was shown by demonstrating that IL-9 neutralization prevents the ability of the vaccine to protect mice against a challenge of live colon carcinoma MC38.CEA cells. In this setting, the authors found that treating mice with cromoglycate, which prevents mast cell activity, or with anti-CD117 antibodies, which deplete mast cells, abrogated the anti-cancer efficacy of the vaccine [26]. Overall, these results illustrate that T_H_9 cells can enhance mast cell activity through IL-9 secretion, resulting in tumor growth prevention (Fig. 1).

## T_H_9 cell-driven activation of adaptive anti-cancer immunity

While IL-9 promotes the activation of innate immune cells like mast cells, which can then contribute to tumor growth prevention, other studies clearly suggest that the anti-tumor activity of IL-9 is not only due to the activation of innate immune effectors. In this regard, upon studying the ability of T_H_9 cell transfer to prevent the development of lung tumor foci in mice injected i.v. with B16 tumor cells, Lu et al. found enhanced leukocyte infiltration in lung tumor tissues [27]. Specifically, increased infiltration with CD4 and CD8 cells as well as dendritic cells (DCs) was noted, thereby suggesting the induction of an adaptive immune response. This hypothesis was further reinforced by the observation that CD44 expression on T cells was upregulated in mice receiving T_H_9 cells. They further found that T_H_9 cells also drove Ccl20/Ccr6-dependent recruitment of DCs to the tumor tissues, thereby driving CD8 T cell activation (Fig. 1). To test whether CD8 T cells were involved in the T_H_9 cell anti-tumor effect, the authors first assessed the frequency of tumor-specific CD8 T cells in mice having received T_H_9 cells. They found increased frequencies of tumor-specific CD8 T cells in the tumor tissues, in contrast to control mice or T_H_1-treated mice. The critical contribution of CD8 T cells in mediating the anti-cancer effects of the transferred T_H_9 cells was eventually confirmed by depleting CD8 T cells using anti-CD8 antibodies. In this setting, the injection of anti-CD8 antibodies nullified the anti-tumor efficacy of T_H_9 cell transfer in vivo. The ability of T_H_9 cell-derived IL-9 to drive the activation of anti-cancer CD8 T cells was further confirmed recently as illustrated by Zhao et al., who found that immunization of tumor-bearing mice with dectin-1-activated DCs induced potent anti-tumor responses that depended on Th9 cells and the IL-9-dependent induction of anti-cancer CD8 T cells [28].

We have also explored the anti-cancer efficacy of T_H_9 cell transfer in vivo in the B16 melanoma model. In agreement with Lu et al., we also found that the anti-cancer effects of T_H_9 cell transfer were dependent on CD8 T cells in vivo. Upon investigating the role of proinflammatory factors on T_H_9 cell differentiation, we found that IL-1β actually enhanced T_H_9 cell induction, as illustrated by increased IL-9 secretion from T_H_9 cells [29]. We also found that IL-1β enhanced IL-21 secretion levels from differentiating T_H_9 cells [29]. These observations relied on the STAT1-dependent activation of the interferon regulatory factor 1 (IRF1) transcription factor, which bound to the IL-9 and IL-21 promoters in differentiating T_H_9 cells. Upon investigating the anti-cancer properties of T_H_9 cells differentiated in the presence of IL-1β, we noted that IL-21 was critical in driving the anti-tumor effects of T_H_9 cells upon adoptive transfer in vivo. IL-21 drove IFN-γ secretion from both NK and CD8 T cells, which were both responsible for tumor elimination. Altogether, these studies suggest that T_H_9 cells trigger IL-9 and IL-21-dependent, CD8-dependent anti-tumor responses that favor tumor elimination. It is noteworthy that beyond IL-9 and IL-21, T_H_9 cell-derived IL-3 could additionally contribute to the induction of adaptive anti-cancer immune responses. It has indeed been reported that T_H_9 cell-derived IL-3 could favor DC survival [30] (Fig. 1).

**Fig. 2:**
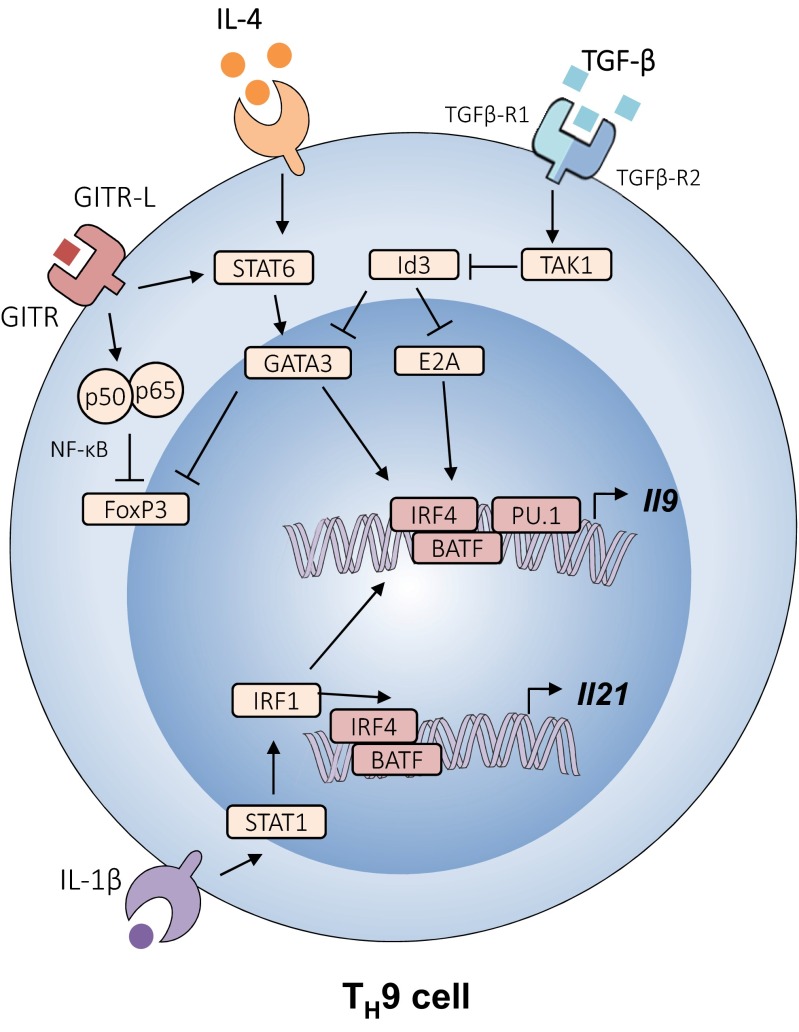
Signaling pathways that promote interleukin-9 and interleukin-21 production

While most of the pioneer studies showing the anti-cancer efficacy of T_H_9 cells relied on adoptive transfer settings, T_H_9 cells were recently shown in two studies to be responsible for anti-cancer immune responses following engagement of glucocorticoid-induced TNFR-related protein (GITR) [31, 32], a costimulatory molecule present on T cells (Fig. 2). By studying the expression of *Il9* in CT26 colon adenocarcinoma tumor-bearing mice treated with an agonistic anti-GITR antibody (DTA-1), Kim et al. found that the engagement of GITR was associated with strong IL-9 expression in the tumor-draining lymph nodes [31]. They next identified T_H_9 cells as the major source of IL-9 in this tumor model in response to DTA-1 treatment. They found that neutralization of IL-9 abrogated the beneficial anti-tumor effect of DTA-1. Importantly, the authors found that DTA-1 also prevent tumor development in an IL-9-dependent manner in non-transplantable models, including a chemical-induced colorectal cancer and the spontaneous K-Ras transgenic mouse model. Kim and colleagues then investigated the role of CD8 T cell responses in the IL-9-dependent anti-cancer efficacy of the DTA-1 treatment. They found that blockade of IL-9 partly prevented the cytotoxic activity of CD8 T cells. Interestingly, they also noted that DTA-1 enhanced IL-21 secretion from CD4 T cells in an IL-9-independent manner and proposed that DTA-1 enhances anti-tumor responses through T_H_9 cell-derived IL-9 and, possibly, IL-21 (Fig. 2). Overall, these results indicate that in the context of lung adenocarcinoma, colon carcinoma, and melanoma, T_H_9 cells promote activation of adaptive anti-tumor immune responses through their secretion of IL-9 and IL-21 (Fig. 1).

### T_H_9 cell cytotoxicity on cancer cells

While it is clear that T_H_9 cells promote the activation of innate and adaptive immune responses, the ability of T_H_9 cells to directly trigger cancer cell death remains debated. Initially, Purwar et al. showed that unlike T_H_0 and T_H_17 cells, T_H_9 cells derived from OT-II transgenic mice, which express a transgenic T cell receptor able to recognize chicken ovalbumin-derived peptides, killed ovalbumin-expressing (B16-OVA) tumor cells. They further noted that T_H_9 cells expressed high levels of granzyme-B and that its inhibition reduced the ability of T_H_9 cells to kill melanoma cells [22]. By contrast, others failed to note any direct cytotoxicity of T_H_9 cells on tumor cells in vitro [27]. Nevertheless, the cytotoxic properties of T_H_9 cells were also shown in a mouse model of squamous cancer [33], suggesting that the cytotoxic activity of T_H_9 cells may be context and tumor specific. Importantly, IL-9 was recently shown to inhibit proliferation of two human melanoma tumor cell lines through upregulation of p21 and TRAIL [34], giving additional impetus to investigate further the direct cytotoxic effects of IL-9 on cancer cells (Fig. 1).

## T_H_9 cell stability in vivo in a cancer setting

The use of an Il9 mouse reporter model suggested that, in a model of acute lung injury induced by papain, IL-9 production was transient in vivo [35]. In line with this, while the antigenic stimulation of mouse T_H_9 cells in vitro resulted in a steady increase in IL-10 secretion, IL-9 levels peaked 3 days following stimulation but then sharply decreased, thereby questioning T_H_9 cell stability [36]. In vivo, T_H_9 cells have accordingly been reported to be unstable in some autoimmune disease models, and T_H_9 cells recovered from inflamed sites or LNs produced predominantly IFN-γ [16, 36, 37]. These observations can likely be attributed to the inflammatory environment, since several cytokines, including IFN-γ or IL-23, can alter T_H_9 cell phenotype or reduce IL-9 production [22, 37–39].

Despite the aforementioned findings, Lu et al. demonstrated in a cancer setting that T_H_9 cells were able to retain their cytokine expression profile in vivo [27]. They observed following adoptive transfer into tumor-bearing mice that T_H_9 cells maintained their IL-9 and IL-10 production in the tumor-draining lung lymph node (LLN) without switching to IFN-γ or IL-17 producers. Interestingly, there were high levels of IFN-γ production in the LLNs of mice receiving T_H_9 cell transfer, indicating that not only was IFN-γ derived from host cells but also that transferred T_H_9 cells induced activation of host immune effector cells. Importantly, IFN-γ and IL-17 were detected in the lungs of T_H_9 cell-transferred mice, whereas these cytokines were absent in the lungs of T_H_1 cell-transferred mice. These results suggest that T_H_9 cells not only induce effector cell activation in the tumor-draining lymph nodes but also recruit them into the tumor site to exert their tumor-killing functions. To further test whether T_H_9 cells maintained their phenotype in vivo, the authors labeled tumor-specific T_H_9 cells with CFSE and transferred them to tumor-bearing mice. On day 4, after transfer, CFSE+ T_H_9 cells retained their production of IL-9 and IL-10, with very low amounts of IFN-γ, TNF-α, and IL-17 secreted, thereby showing that transferred T_H_9 cells maintained their IL-9 and IL-10 cytokine production and did not convert to T_H_1 or T_H_17 cell subsets.

We have also studied the fate of T_H_9 cells in tumor-bearing mice in vivo to assess their stability. For this, we transferred IL-1β-induced CD45.2^+^ OT-II T_H_9 cells into CD45.1^+^ mice bearing B16-OVA tumors. We sorted CD45.2^+^ cells from lung-draining lymph nodes and analyzed their gene-expression profiles 6 days after transfer. Importantly, CD45.2^+^ cells maintained their expression of T_H_9-related genes such as *Irf4*, *Spi1*, *Il9*, and *Il21* and did not gain expression of T_H_1-related genes after transfer [29]. These data indicated that IL-1β-induced T_H_9 cells maintained their transcriptional program in vivo. We speculate that the IL-1β-induced expression of IRF1 contributes to stabilize the T_H_9 cell transcriptional program. Altogether, this suggests that in a cancer setting, T_H_9 cells are capable of maintaining IL-9- and IL-21-secreting potential, at least long enough to carry out their anti-cancer functions in vivo. These observations may prove relevant in the context of vaccination because a protective T_H_9 cell-specific anti-cancer response could be induced through immunization against tumor antigens [26]. Whether vaccination protocols could be optimized to induce stable and specific T_H_9 memory anti-tumor cells remains to be determined.

## The role of T_H_9 cells in human cancers

The suggested superiority of T_H_9 cells in controlling tumor progression in vivo in mice upon adoptive transfer and the detection of memory T_H_9 cells in healthy human blood and skin underscore the potential relevance of this subset in cancer immunotherapy [22]. Schlapbach and colleagues further characterized human IL-9-producing T cells [40]. In humans, IL-9 is primarily produced by a discrete and stable population of T cells. Unlike human and mouse T cells generated by differentiation from naïve cells in vitro, human T_H_9 cells isolated from human blood and tissues lacked expression of other T_H_ lineage cytokines; co-produced TNF-α, granzyme B; and lacked FOXP3 expression. Both the circulating and the in vitro differentiated T_H_9 cells lacked IL-10 production, a feature that distinguishes them from mouse T_H_9 cells. To sort human memory skin and gut tropic T_H_ cells, Clark and colleagues used an elegant approach based on the expression of the skin-homing receptor cutaneous lymphocyte antigen (CLA) and the gut-homing receptor integrin α_4_β_7_. In healthy adults, T_H_9 cells were found primarily among the CLA^+^ skin-homing effector T cell population and were present in healthy human skin but were absent from human small intestine and lung [40]. In line with their role in inflammation and skin tropism, T_H_9 cells were proposed to be involved in the pathogenesis of respiratory epithelial adenomatoid hamartoma (REAH). Accordingly, in REAH tissue, T_H_9 cell population was expanded, the synthesis of IL-9-encoding mRNA upregulated, and IL-9 secretion increased, suggesting that T_H_9 cells play a role in the pathogenesis of the disease [41].

The direct involvement of human T_H_9 cells in malignancy remains elusive. T_H_9 cell numbers were found to be increased in malignant pleural effusion (MPE) when compared with blood [42]. Accordingly, Shi et al. reported that the recruitment of T_H_9 cells into MPE could be induced by pleural CCL20 expression and that the majority of T_H_9 cells expressed a high level of CCR6 on their surface and displayed an effector memory phenotype characterized by high expression of CD45RO and very low levels of CD45RA and CD62L [43]. In this context, it was shown that the increase in pleural T_H_9 cells predicted reduced survival in patients with MPE [42]. These findings contrast with other studies suggesting anti-cancer functions for T_H_9 cells. Specifically, T_H_9 cells were found in metastatic lesions of human patients with melanoma. The subsequent analysis of TILs extracted from these metastatic lesions revealed detectable levels of IL-9-producing CD4 T cells, lacking IFNγ, IL-4, and IL-17 production. However, T_H_9 cells were less abundant in the TILs from these melanoma lesions compared to healthy skin, and their IL-9 secretion was also reduced [22], possibly suggesting that human T_H_9 cells are protective against melanoma development. Interestingly, IL-9 was recently shown to promote the survival and function of human melanoma-infiltrating CD4+CD8+ double-positive T cells [44] (Fig. 1), lending further support to the hypothesis that IL-9 may amplify anti-tumor immune responses by promoting T cell fitness in human melanoma. Accordingly, single-nucleotide polymorphisms in the IL-9 gene are associated with an increased risk of cutaneous malignant melanoma [45]. These findings suggest that strategies favoring the generation of T_H_9 cell-mediated immune responses may have an important role in the treatment of melanoma in humans. In the light of the latest advances on T_H_9 cell anti-tumor properties [28, 32], the use of dendritic cell-based therapeutic cancer vaccines could be considered to generate a T_H_9 cell response in cancer patients. Indeed, DC-expressing GITR-L [46] or activated via the dectin-1 signaling pathway [28] may generate a T_H_9 cell anti-tumor response that ultimately results in a clinical benefit in cancer patients.

## Conclusions and prospects

T_H_9 cells have an important role in tumor immunity that seems to be largely due to their ability to secrete IL-9 and IL-21 cytokines. IL-9 is a pleiotropic cytokine that can have direct anti-tumor effects or can indirectly influence tumor growth by enhancing immune responses. IL-21 likewise strongly supports the activation of adaptive anti-cancer immunity. Whereas growing evidence supports the potential relevance of T_H_9 cells to cancer immunity especially in the context of adoptive cell therapy strategies, a complete understanding of the physiological conditions that lead to the generation and expansion of this particular helper T cell subset is still lacking [47, 48]. Unraveling the mechanisms that underpin therapeutic resistance to T_H_9 cells in tumor-bearing hosts and/or combining T_H_9 cell-based therapies with immunomodulators or chemotherapy could promote potent and long-lasting anti-tumor immunity [49]. Finally, determining whether the anti-cancer properties of human T_H_9 cells extend beyond the melanoma setting is essential.

T_H_9 cells harbor strong anti-cancer properties in the context of solid tumors. Through the production of granzyme-B, T_H_9 cells trigger cancer cell death [22]. T_H_9-derived IL-9 leads to upregulation of p21 and TRAIL, thus inducing tumor cell cycle arrest and apoptosis upon activation of IL-9R [34]. T_H_9 cells are also able to promote the activation of innate cells [22, 23]. Accordingly, IL-9 enhances the anti-tumor activity of mast cells [22, 25, 26]. T_H_9 cells enhance the induction of anti-tumor adaptive immune responses. IL-9 promotes Ccl20 expression from epithelial cells, thus driving Ccl20/Ccr6-dependent leucocyte and DC recruitment [27]. Moreover, T_H_9 cell-derived IL-9 enhances IFN-γ secretion from CD8 T cells [28], while T_H_9-derived IL-3 can promote DC survival [30]. IL-21 drives IFN-γ secretion from both NK and CD8 T cells, which are both responsible for tumor elimination [29].

The regulation of T_H_9 cell differentiation by the transcription factor Id3 influences anti-melanoma immunity in an IL-9-dependent fashion [23]. Mechanistically, TGF-β1 and IL-4 downregulate Id3 expression through TAK1 kinase. This reduction in Id3 expression enhances binding of the transcription factors E2A and GATA-3 to the Il9 promoter region, which promotes Il9 transcription [23]. Activation of GITR by its ligand enhances IL-9 expression in a STAT6- and NF-κB-dependent manner, followed by anti-cancer immune responses [31, 32]. In the presence of IL-1β, IRF1 expression is upregulated in a STAT1-dependent fashion. Subsequently, IRF1 binds to the *Il9* and *Il21* promoters in T_H_9 cells, which increases the secretion of both IL-9 and IL-21 [29].
